# Supplementary material for: Heparin-based hydrogel scaffolding alters the transcriptomic profile and increases the chemoresistance of MDA-MB-231 triple-negative breast cancer cells
Source: Biomater Sci. 2020 Feb 13;8(10):2786–96. doi: 10.1039/c9bm01481k (PMC7497406; doi:10.1039/c9bm01481k)
Supplement: Supplementary file 2 [file BM-008-C9BM01481K-s002.zip › Supplementary File 4/HP-B hydrogel + EGF (s)vControl/Pathways/my_analysis.Gsea.1545201170987/HALLMARK_UV_RESPONSE_DN.html]

Details for gene set HALLMARK\_UV\_RESPONSE\_DN[GSEA]

|  || Dataset | expr.class.cls#HYDEGF\_versus\_CONTROL.class.cls#HYDEGF\_versus\_CONTROL\_repos |
| Phenotype | class.cls#HYDEGF\_versus\_CONTROL\_repos |
| Upregulated in class | CONTROL |
| GeneSet | HALLMARK\_UV\_RESPONSE\_DN |
| Enrichment Score (ES) | -0.17356294 |
| Normalized Enrichment Score (NES) | -0.76652193 |
| Nominal p-value | 0.9660194 |
| FDR q-value | 0.9355786 |
| FWER p-Value | 1.0 |
Table: GSEA Results Summary

  

Fig 1: Enrichment plot: HALLMARK\_UV\_RESPONSE\_DN      
 Profile of the Running ES Score & Positions of GeneSet Members on the Rank Ordered List

  

| PROBE | DESCRIPTION (from dataset) | GENE SYMBOL | GENE\_TITLE | RANK IN GENE LIST | RANK METRIC SCORE | RUNNING ES | CORE ENRICHMENT || 1 | RBPMS | na |  |  | 509 | 2.108 | -0.0058 | Yes |
| 2 | SYNE1 | na |  |  | 589 | 2.028 | 0.0101 | Yes |
| 3 | SMAD7 | na |  |  | 606 | 2.006 | 0.0291 | Yes |
| 4 | DLC1 | na |  |  | 642 | 1.960 | 0.0466 | Yes |
| 5 | PRKCA | na |  |  | 865 | 1.773 | 0.0525 | Yes |
| 6 | RUNX1 | na |  |  | 1207 | 1.566 | 0.0501 | Yes |
| 7 | PTPN21 | na |  |  | 1290 | 1.519 | 0.0608 | Yes |
| 8 | ADORA2B | na |  |  | 1353 | 1.493 | 0.0723 | Yes |
| 9 | VAV2 | na |  |  | 1446 | 1.450 | 0.0819 | Yes |
| 10 | PRKCE | na |  |  | 1676 | 1.366 | 0.0834 | Yes |
| 11 | PTPRM | na |  |  | 1775 | 1.337 | 0.0914 | Yes |
| 12 | PAPD7 | na |  |  | 1842 | 1.313 | 0.1010 | Yes |
| 13 | COL1A1 | na |  |  | 1873 | 1.303 | 0.1123 | Yes |
| 14 | MAGI2 | na |  |  | 1942 | 1.280 | 0.1214 | Yes |
| 15 | COL5A2 | na |  |  | 2434 | 1.144 | 0.1069 | Yes |
| 16 | MTA1 | na |  |  | 2565 | 1.111 | 0.1111 | Yes |
| 17 | DYRK1A | na |  |  | 2643 | 1.092 | 0.1179 | Yes |
| 18 | ARHGEF9 | na |  |  | 2806 | 1.051 | 0.1198 | Yes |
| 19 | DBP | na |  |  | 2838 | 1.044 | 0.1285 | Yes |
| 20 | F3 | na |  |  | 2957 | 1.016 | 0.1323 | Yes |
| 21 | ERBB2 | na |  |  | 3000 | 1.004 | 0.1400 | Yes |
| 22 | WDR37 | na |  |  | 3019 | 1.000 | 0.1490 | Yes |
| 23 | ABCC1 | na |  |  | 3065 | 0.988 | 0.1564 | Yes |
| 24 | DUSP1 | na |  |  | 3230 | 0.954 | 0.1572 | Yes |
| 25 | CDK13 | na |  |  | 3389 | 0.925 | 0.1581 | Yes |
| 26 | FHL2 | na |  |  | 3633 | 0.879 | 0.1540 | Yes |
| 27 | SLC7A1 | na |  |  | 3784 | 0.850 | 0.1546 | Yes |
| 28 | YTHDC1 | na |  |  | 3943 | 0.822 | 0.1544 | Yes |
| 29 | SFMBT1 | na |  |  | 4171 | 0.787 | 0.1503 | Yes |
| 30 | MYC | na |  |  | 4314 | 0.764 | 0.1504 | Yes |
| 31 | GRK5 | na |  |  | 4574 | 0.723 | 0.1440 | Yes |
| 32 | IRS1 | na |  |  | 4622 | 0.716 | 0.1486 | Yes |
| 33 | DLG1 | na |  |  | 4883 | 0.670 | 0.1416 | Yes |
| 34 | SCAF8 | na |  |  | 4992 | 0.653 | 0.1424 | Yes |
| 35 | PIK3R3 | na |  |  | 5161 | 0.627 | 0.1398 | Yes |
| 36 | MAPK14 | na |  |  | 5197 | 0.620 | 0.1441 | Yes |
| 37 | CDON | na |  |  | 5220 | 0.618 | 0.1490 | Yes |
| 38 | CDC42BPA | na |  |  | 5261 | 0.611 | 0.1530 | Yes |
| 39 | RXRA | na |  |  | 5268 | 0.611 | 0.1587 | Yes |
| 40 | SCHIP1 | na |  |  | 5310 | 0.603 | 0.1625 | Yes |
| 41 | BDNF | na |  |  | 5346 | 0.597 | 0.1666 | Yes |
| 42 | PIK3CD | na |  |  | 5415 | 0.589 | 0.1688 | Yes |
| 43 | PRKAR2B | na |  |  | 5538 | 0.571 | 0.1681 | Yes |
| 44 | COL1A2 | na |  |  | 5782 | 0.534 | 0.1606 | Yes |
| 45 | NR3C1 | na |  |  | 5797 | 0.533 | 0.1651 | Yes |
| 46 | FZD2 | na |  |  | 5808 | 0.531 | 0.1699 | Yes |
| 47 | CAP2 | na |  |  | 5864 | 0.523 | 0.1722 | Yes |
| 48 | SMAD3 | na |  |  | 6056 | 0.496 | 0.1671 | No |
| 49 | NR1D2 | na |  |  | 6130 | 0.485 | 0.1680 | No |
| 50 | MIOS | na |  |  | 6449 | 0.442 | 0.1557 | No |
| 51 | SYNJ2 | na |  |  | 6666 | 0.415 | 0.1485 | No |
| 52 | CACNA1A | na |  |  | 6812 | 0.395 | 0.1448 | No |
| 53 | ID1 | na |  |  | 6833 | 0.393 | 0.1476 | No |
| 54 | IGF1R | na |  |  | 6976 | 0.375 | 0.1439 | No |
| 55 | BCKDHB | na |  |  | 6997 | 0.371 | 0.1465 | No |
| 56 | NOTCH2 | na |  |  | 7202 | 0.343 | 0.1392 | No |
| 57 | PDGFRB | na |  |  | 7303 | 0.329 | 0.1372 | No |
| 58 | TGFBR3 | na |  |  | 7326 | 0.326 | 0.1393 | No |
| 59 | LDLR | na |  |  | 8020 | 0.241 | 0.1053 | No |
| 60 | INPP4B | na |  |  | 8021 | 0.241 | 0.1077 | No |
| 61 | ATP5S | na |  |  | 8062 | 0.235 | 0.1079 | No |
| 62 | ATP2C1 | na |  |  | 8173 | 0.230 | 0.1044 | No |
| 63 | TJP1 | na |  |  | 8311 | 0.208 | 0.0993 | No |
| 64 | NFKB1 | na |  |  | 8333 | 0.205 | 0.1002 | No |
| 65 | MGLL | na |  |  | 8440 | 0.196 | 0.0966 | No |
| 66 | RND3 | na |  |  | 8466 | 0.193 | 0.0972 | No |
| 67 | MMP16 | na |  |  | 8631 | 0.167 | 0.0903 | No |
| 68 | DDAH1 | na |  |  | 8750 | 0.158 | 0.0856 | No |
| 69 | KALRN | na |  |  | 9077 | 0.118 | 0.0697 | No |
| 70 | PRDM2 | na |  |  | 9115 | 0.112 | 0.0689 | No |
| 71 | NFIB | na |  |  | 9178 | 0.105 | 0.0666 | No |
| 72 | MAP2K5 | na |  |  | 9350 | 0.082 | 0.0585 | No |
| 73 | APBB2 | na |  |  | 9849 | 0.029 | 0.0327 | No |
| 74 | NIPBL | na |  |  | 10052 | 0.012 | 0.0222 | No |
| 75 | AKT3 | na |  |  | 10132 | 0.001 | 0.0180 | No |
| 76 | GCNT1 | na |  |  | 10315 | -0.025 | 0.0087 | No |
| 77 | SNAI2 | na |  |  | 10596 | -0.050 | -0.0054 | No |
| 78 | BMPR1A | na |  |  | 10633 | -0.057 | -0.0068 | No |
| 79 | NEK7 | na |  |  | 10732 | -0.069 | -0.0112 | No |
| 80 | EFEMP1 | na |  |  | 10740 | -0.071 | -0.0109 | No |
| 81 | CITED2 | na |  |  | 10743 | -0.071 | -0.0103 | No |
| 82 | CAV1 | na |  |  | 11391 | -0.145 | -0.0428 | No |
| 83 | ATRX | na |  |  | 11758 | -0.197 | -0.0600 | No |
| 84 | KCNMA1 | na |  |  | 11905 | -0.214 | -0.0655 | No |
| 85 | ATXN1 | na |  |  | 12145 | -0.236 | -0.0757 | No |
| 86 | ZMIZ1 | na |  |  | 12259 | -0.254 | -0.0792 | No |
| 87 | MAP1B | na |  |  | 12370 | -0.270 | -0.0823 | No |
| 88 | ADD3 | na |  |  | 12405 | -0.273 | -0.0814 | No |
| 89 | LPAR1 | na |  |  | 12426 | -0.275 | -0.0797 | No |
| 90 | SIPA1L1 | na |  |  | 12712 | -0.315 | -0.0915 | No |
| 91 | FYN | na |  |  | 12769 | -0.326 | -0.0912 | No |
| 92 | ATP2B4 | na |  |  | 13238 | -0.376 | -0.1120 | No |
| 93 | PEX14 | na |  |  | 13302 | -0.386 | -0.1115 | No |
| 94 | MET | na |  |  | 13324 | -0.390 | -0.1088 | No |
| 95 | ACVR2A | na |  |  | 13516 | -0.416 | -0.1147 | No |
| 96 | PIAS3 | na |  |  | 13917 | -0.474 | -0.1310 | No |
| 97 | SPOP | na |  |  | 13989 | -0.488 | -0.1299 | No |
| 98 | LAMC1 | na |  |  | 14471 | -0.552 | -0.1496 | No |
| 99 | RASA2 | na |  |  | 14724 | -0.593 | -0.1570 | No |
| 100 | RGS4 | na |  |  | 15008 | -0.634 | -0.1656 | No |
| 101 | ATP2B1 | na |  |  | 15051 | -0.643 | -0.1614 | No |
| 102 | MT1E | na |  |  | 15125 | -0.658 | -0.1587 | No |
| 103 | ATRN | na |  |  | 15292 | -0.685 | -0.1607 | No |
| 104 | MRPS31 | na |  |  | 15539 | -0.731 | -0.1663 | No |
| 105 | PPARG | na |  |  | 15629 | -0.748 | -0.1636 | No |
| 106 | CDKN1B | na |  |  | 15677 | -0.758 | -0.1586 | No |
| 107 | CELF2 | na |  |  | 15756 | -0.775 | -0.1550 | No |
| 108 | PDLIM5 | na |  |  | 15884 | -0.799 | -0.1538 | No |
| 109 | SLC22A18 | na |  |  | 15992 | -0.817 | -0.1513 | No |
| 110 | SRI | na |  |  | 16360 | -0.913 | -0.1615 | No |
| 111 | PTEN | na |  |  | 16477 | -0.941 | -0.1583 | No |
| 112 | PHF3 | na |  |  | 16574 | -0.962 | -0.1538 | No |
| 113 | GJA1 | na |  |  | 16597 | -0.968 | -0.1454 | No |
| 114 | BHLHE40 | na |  |  | 16798 | -1.027 | -0.1457 | No |
| 115 | HAS2 | na |  |  | 16873 | -1.047 | -0.1393 | No |
| 116 | INSIG1 | na |  |  | 17226 | -1.153 | -0.1463 | No |
| 117 | NRP1 | na |  |  | 17279 | -1.174 | -0.1374 | No |
| 118 | SDC2 | na |  |  | 17642 | -1.310 | -0.1435 | No |
| 119 | VLDLR | na |  |  | 17779 | -1.371 | -0.1370 | No |
| 120 | DAB2 | na |  |  | 17915 | -1.448 | -0.1298 | No |
| 121 | PMP22 | na |  |  | 18110 | -1.553 | -0.1246 | No |
| 122 | ICA1 | na |  |  | 18335 | -1.703 | -0.1195 | No |
| 123 | TGFBR2 | na |  |  | 18371 | -1.729 | -0.1043 | No |
| 124 | AGGF1 | na |  |  | 18372 | -1.730 | -0.0872 | No |
| 125 | ANXA4 | na |  |  | 18571 | -1.892 | -0.0788 | No |
| 126 | ITGB3 | na |  |  | 18981 | -2.535 | -0.0752 | No |
| 127 | TFPI | na |  |  | 19039 | -2.696 | -0.0515 | No |
| 128 | PTGFR | na |  |  | 19066 | -2.792 | -0.0253 | No |
| 129 | LTBP1 | na |  |  | 19148 | -3.253 | 0.0026 | No |
Table: GSEA details [plain text format]

  

Fig 2: HALLMARK\_UV\_RESPONSE\_DN      
 Blue-Pink O' Gram in the Space of the Analyzed GeneSet

  

Fig 3: HALLMARK\_UV\_RESPONSE\_DN: Random ES distribution      
 Gene set null distribution of ES for **HALLMARK\_UV\_RESPONSE\_DN**
